# Comparing Injection Methods of Botulinum Toxin A for Cervical Dystonia: A Systematic Review

**DOI:** 10.3390/life15060920

**Published:** 2025-06-06

**Authors:** Hristo Shipkov, Petar Uchikov, Abdulrahman Imran, Zain Ul Hassan, Ivan Grozdev, Krasimir Kraev, Maria Kraeva, Nina Koleva, Maria Bozhkova, Stanislav Karamitev

**Affiliations:** 1 Department of Special Surgery, Faculty of Medicine, Medical University of Plovdiv, 4002 Plovdiv, Bulgaria; cshipkov@gmail.com (H.S.); puchikov@yahoo.com (P.U.); 2 Division of Plastic Surgery, Medical University of Plovdiv, 4000 Plovdiv, Bulgaria; 3 Faculty of Medicine, Medical University of Plovdiv, 4000 Plovdiv, Bulgaria; imran.ar057@gmail.com (A.I.); zulhassan2002@gmail.com (Z.U.H.); 4 Department of Neurology, University Hospital Kaspela, 4000 Plovdiv, Bulgaria; mc.avangard.ig@gmail.com; 5 Department of Propedeutics of Internal Diseases, Medical Faculty, Medical University of Plovdiv, 4002 Plovdiv, Bulgaria; 6 Department of Otorhinolaryngology, Medical Faculty, Medical University of Plovdiv, 4002 Plovdiv, Bulgaria; kraevamaria93@gmail.com; 7 Specialty “Assistant Pharmacist”, Medical College, Medical University of Plovdiv, 4000 Plovdiv, Bulgaria; nina.koleva@mu-plovdiv.bg; 8 Department of Orthopedics and Traumatology, Medical Faculty, Medical University of Plovdiv, 4000 Plovdiv, Bulgaria; mariya.bozhkova@mu-plovdiv.bg (M.B.); dokstanly@yahoo.com (S.K.)

**Keywords:** cervical dystonia, botulinum toxin injection, ultrasound and EMG guidance

## Abstract

Background: Cervical dystonia (CD) is a chronic neurological disorder characterized by involuntary neck muscle contractions, leading to abnormal head postures, pain, and functional impairment. Botulinum toxin type A (BoNT-A) remains the treatment of choice, but its efficacy is highly dependent on injection accuracy. Various techniques, including palpation-guided, ultrasound-guided, and electromyography-guided (EMG), have been developed to optimize delivery, each with distinct advantages and limitations. Methods: A systematic search of PubMed and Scopus was conducted up until 30 December 2024, using defined keywords related to BoNT-A, CD, and injection techniques. Studies were included if they reported clinical outcomes of BoNT-A injection methods in adult CD patients. Data on efficacy, safety, accuracy, and muscle targeting were extracted and synthesized. Results: Seven studies comprising 239 patients were included: two randomized controlled trials, one retrospective study, one cohort study, one systematic review, one literature review, and one cadaveric study. The most common CD subtype was torticollis/torticaput (49.79%). Frequently targeted muscles included the trapezius (56.9%), levator scapulae (51.7%), and splenius capitis (48.3%). Ultrasound guidance consistently demonstrated superior injection accuracy and reduced adverse effects due to real-time anatomical visualization. EMG-guided techniques showed advantages in identifying dystonic muscles, especially when anatomy was unclear. In contrast, palpation-guided injections were less accurate and suitable only for superficial muscles. Dosing varied by product, with mean doses of 117–118 units for onabotulinumtoxinA and incobotulinumtoxinA, and 405 units for abobotulinumtoxinA. Adverse events were generally mild, including local discomfort, dysphagia, and transient muscle weakness. Conclusions: Ultrasound- and EMG-guided injections enhance the precision, safety, and efficacy of BoNT-A therapy for CD compared to anatomy-guided techniques. While ultrasound guidance improves anatomical accuracy, EMG remains valuable for functionally identifying dystonic muscles. Integration of both may offer optimal outcomes. However, further high-quality, standardized trials are needed to definitively establish best practices.

## 1. Introduction

Cervical dystonia, also known as spasmodic torticollis, is a chronic neurological disorder characterized by involuntary contractions of the neck muscles, leading to abnormal postures, tremors, and significant pain [1]. Reported prevalence rates range from 28 to 183 cases per million, while incidence estimates fall between 8 and 12 new cases per million person-years, depending on the population studied and the methodology used. The condition typically presents in the fourth to fifth decade of life, with a mean age at onset of approximately 42 years. Across epidemiological studies, CD shows a notable gender disparity, with an overall female-to-male ratio of approximately 1.7:1 [2].

This condition impacts physical function, psychological well-being, and overall quality of life, necessitating effective treatment strategies. While oral medications such as anticholinergics, muscle relaxants, and benzodiazepines are sometimes used as initial treatments, they are often limited by systemic side effects, including sedation, cognitive impairment, and dry mouth, and typically offer only modest relief. Consequently, BoNT has emerged as another option in the treatment of cervical dystonia due to its ability to reduce muscle overactivity with a localized mechanism of action and fewer systemic side effects [3]. Despite its established efficacy, the success of BoNT treatment depends largely on the precision of its injection technique, which is pivotal to optimizing therapeutic outcomes, as the product must be injected precisely into the affected muscle. In this context, several methods have been used to mark the muscle for injection, including anatomy/palpation-guided, ultrasound-guided, PET/CT-assisted, EMG-guided, and kinematic-guided techniques [4]. Each method has its advantages and limitations. For instance, palpation-guided techniques rely on clinical expertise and anatomical knowledge, whereas imaging and electrophysiological guidance, such as ultrasound and EMG, provide more objective targeting of the involved muscles but require additional training [5]. Emerging technologies such as PET/CT-assisted and kinematic-guided injections further expand the scope of precision but may come with increased complexity and resource requirements [6]. While individual studies have explored these methods, a comprehensive comparison of their effectiveness, safety, and clinical outcomes is lacking. Understanding the relative merits of each approach is crucial for clinicians to make informed decisions tailored to patient needs and clinical settings. The diverse clinical presentations of cervical dystonia, ranging from torticollis to retrocollis, introduce significant complexity into therapeutic decision-making. Effective management requires an individualized approach that accounts for the specific muscles involved, the severity of symptoms, disease duration, and the patient’s tolerance to treatment. These considerations highlight the importance of precision, particularly in the administration of botulinum toxin A (BoNT-A). Advances in imaging and neurophysiological techniques have meaningfully influenced BoNT-A delivery. The integration of high-resolution ultrasound and EMG allows clinicians to visualize or confirm dystonic muscle activity in real time, enabling more targeted and personalized injection strategies. These tools not only enhance treatment accuracy but also hold promise for minimizing complications, such as unintended toxin diffusion to adjacent, unaffected muscles. Functional imaging and electrophysiological markers are becoming increasingly important in the treatment landscape of cervical dystonia. Techniques like PET, SPECT, and advanced fMRI are being used more often to explore the underlying neurophysiology of the disorder [7]. These imaging tools may help pinpoint dystonic muscle activity at the central level, offering a more refined basis for selecting target muscles during BoNT-A injections. Looking ahead, such modalities could complement current anatomical and functional approaches, paving the way for even more personalized and precise treatment strategies. Nevertheless, there is still notable variability in clinical practice, with technique selection often driven more by individual physician preference and available resources than by uniform evidence-based standards. This review aims to address that inconsistency by critically examining the existing literature on injection methodologies and providing insight into their relative clinical value in the management of cervical dystonia.

## 2. Materials and Methods

A systematic literature search was performed using Pubmed (MEDLINE) and Scopus with the following search terms: (“OnabotulinumtoxinA” OR “AbobotulinumtoxinA” OR “Botulinum Toxin, A”) AND (“Cervical Dystonia” OR “Spasmodic Torticollis”) AND (“Injection Methods” OR “Injection Techniques” OR “Administration, Intramuscular” OR “Palpation” OR “EMG” OR “Ultrasound” OR “Electromyography” OR “Guided Injection Methods” OR “Anatomic Landmark” OR “Electrical Stimulation” OR “Fluoroscopy”). The search was restricted to studies published between 1980 and the present day and was filtered to only include studies published in English. The last search was performed on 30 December 2024. We included studies involving adult patients diagnosed with cervical dystonia who received botulinum toxin A treatments, specifically onabotulinumtoxinA (Botox^®^, AbbVie Inc., North Chicago, IL, USA), abobotulinumtoxinA (Dysport^®^, Ipsen Biopharm Ltd., Wrexham, Wales, UK), or incobotulinumtoxinA (Xeomin^®^, Merz Pharmaceuticals GmbH, Frankfurt am Main, Germany). Eligible studies comprised original research such as prospective and retrospective studies, as well as meta-analyses, which evaluated or compared specific injection techniques. Studies were excluded if they involved pediatric populations, were case reports or case series, or did not report outcomes related to injection methods. These criteria are detailed in Table 1.

**Figure 1:**
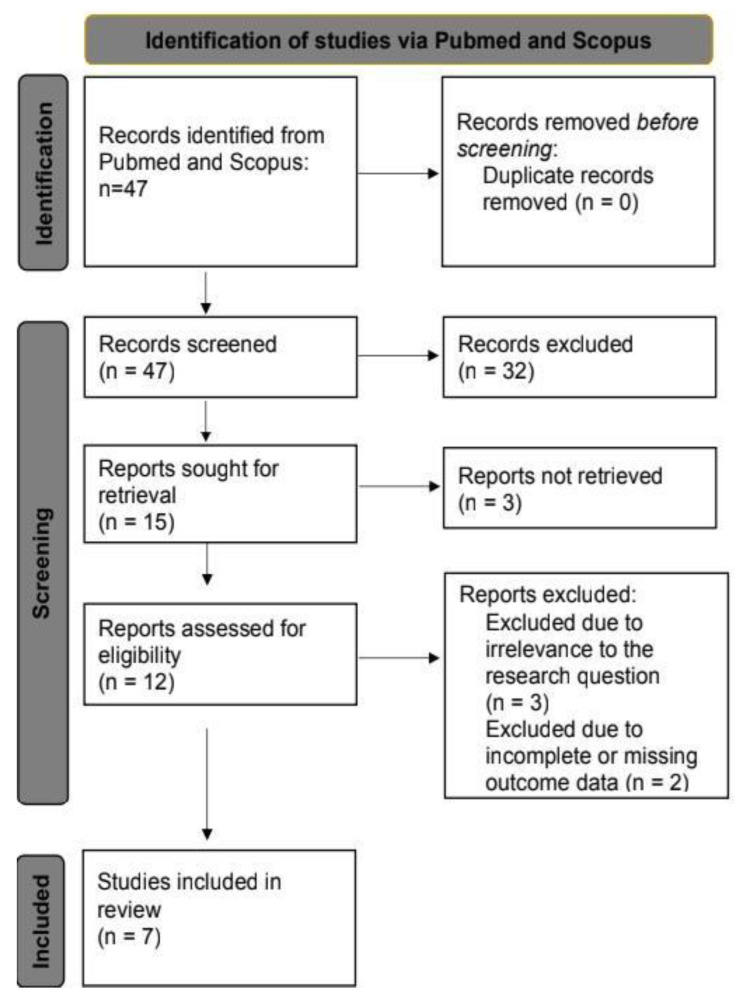
PRISMA flow diagram of study selection. Figure Legends: The PRISMA 2020 flow diagram was generated using the online tool available at the PRISMA Statement website (https://www.prisma-statement.org/). This tool is based on the PRISMA 2020 guidelines developed by Page et al. (2021). This PRISMA flow diagram illustrates the study selection process for this systematic review. It outlines the number of records identified through database searches, the number of studies screened, and the reasons for exclusion at various stages, including duplicates, irrelevant topics, and failure to meet inclusion criteria. It also presents the final number of studies included in the qualitative and quantitative analyses. This systematic approach ensures transparency and reproducibility in the study selection process.

The initial search retrieved 47 studies. Titles and abstracts were screened for relevance based on the inclusion and exclusion criteria. Full-text reviews were conducted for studies deemed potentially eligible. Seven studies met the inclusion criteria and were included in the final synthesis. This process is illustrated in the PRISMA flow diagram (Figure 1).

The primary outcomes of interest included the comparative efficacy of injection methods, safety profiles, and clinical outcomes (e.g., symptom reduction, patient-reported outcomes). Data extraction focused on study characteristics (e.g., sample size, methodology, injection methods used) and reported results. Ethical approval was not required, as this study involved secondary analysis of published data.

Several injection techniques were identified in this search, including injections with anatomy guidance, EMG guidance, polymyographic EMG guidance, and ultrasound guidance. Anatomy-guided injections rely solely on palpation and surface anatomical landmarks to identify and inject target muscles. In contrast, EMG-guided injections use electrical recordings to confirm muscle activity, helping to identify dystonic muscles during rest or voluntary movement. Polymyographic EMG-guided injections expand on this approach by recording from multiple muscles simultaneously, allowing clinicians to assess muscle co-contractions and compensatory patterns in more complex dystonic postures. Finally, ultrasound-guided injections enable real-time visualization of soft tissues, vessels, and muscle planes, particularly for deep or anatomically obscured muscles. Each of these techniques offers distinct advantages and limitations, and their selection often depends on clinician expertise, available resources, and the complexity of the patient’s presentation.

A structured risk of bias assessment was performed for all seven included studies using validated tools appropriate to their design. Randomized controlled trials were evaluated with the Cochrane Risk of Bias 2.0 (RoB 2) tool (version dated 22 August 2019, Cochrane Collaboration, London, UK), while non-randomized observational studies were assessed using the ROBINS-I tool (Cochrane Collaboration, London, UK). The included systematic reviews were appraised using the AMSTAR 2 checklist (September 2017, AMSTAR Research Group, Canada). The randomized controlled trials demonstrated rigorous outcome measurement and appropriate blinding; however, incomplete reporting of randomization methods and lack of protocol registration represent critical methodological gaps that should be addressed in future trials. The non-randomized studies provided valuable real-world insights despite inherent challenges. Most clearly defined interventions and reported minimal missing data; nonetheless, confounding, absence of statistical adjustment, and retrospective designs resulted in an overall moderate risk of bias. The systematic reviews offered clinically relevant perspectives but underscored the need for more standardized and transparent research methodologies to enhance reproducibility and reduce bias. By synthesizing the best available evidence and transparently acknowledging study limitations, this review supports informed clinical decision-making and delineates priorities for future high-quality studies to optimize treatment strategies in cervical dystonia.

This review intentionally focused on primary research studies that directly compared or described injection techniques for BoNT-A in cervical dystonia, with broader reviews and case series being excluded. While this approach limited the total number of included studies, it allowed for a more rigorous appraisal of the current clinical techniques and outcomes reported in original research.

## 3. Results

A total of seven studies were included in this systematic review based on the inclusion criteria, comprising 239 patients diagnosed with cervical dystonia. These studies were published between 2006 and 2023. The study designs varied, with two being randomized controlled trials [8,9], one retrospective cross-sectional study [10], one prospective cohort study [11]. Additionally, one systematic review [12], one literature review [6], and one cadaveric study [13] were identified during the selection process. The systematic review was included for qualitative synthesis but not for pooled statistical analysis, as it summarized findings from multiple studies without providing new patient-level data. The literature review was excluded from this review due to its narrative nature and lack of original quantitative data [6]. The cadaveric study involved 20 specimens and whilst relevant for anatomical insights, it was also excluded from the pooled analysis as it did not involve live patients or clinical outcomes [13]. The randomized controlled trials investigated different injection methods, including anatomy-guided and ultrasound-guided methods. Sample sizes ranged from 56 to 105 patients per trial. Follow-up durations were 1 month, 3 months, and 6 months [8,9]. The prospective cohort study investigated EMG-guided injections among 20 patients. Follow-up durations were 15 and 21 days after injection [11]. In the retrospective cross-sectional study, ultrasound-guided injection was investigated using 58 patients. Across the three studies that reported gender distribution (*n* = 219), the majority of patients were female (121/219, 55.3%), while males comprised 44.7% (98/219) [10]. Age data were available for two studies (*n* = 114), with a weighted mean age of 60.0 years [9,10]. The overall reported age range across studies was 22 to 81 years, though one study provided only an age range (22–57 years) [11], and another did not report age or gender data [11]. Four studies reported cervical dystonia subtypes (*n* = 239); the most common presentation was torticollis/torticaput, affecting 49.79% (119/239) of patients. This information is detailed in Table 2, with summaries of all included studies shown in Table 3.

Head tremor was reported in 51.4% (65/126) of patients from the two studies that mentioned this feature [9,10]. Data on injected muscles were reported in two studies (*n* = 114 patients) [9,10]. Table 4 displays the most frequently targeted muscles:

In the study by A Kreisler [9], 2020, a total of 264 muscles were injected with anatomy guidance, predominantly first-layer muscles (e.g., splenius capitis, SCM, trapezius, levator scapulae, scalenus medius), with second- and third-layer muscles (e.g., semispinalis capitis, semispinalis cervicis) rarely targeted. The mean number of injections per muscle was 1.3 [9]. Dosing data were provided for Máté Szabó, 2023 [10], Table 5, showing the following mean doses:

The mean disease duration in A Kreisler, 2020 was reported as 18.6 ± 11.9 years [9]. Máté Szabó, 2023 indicated a disease duration ranging from 6 months to 10 years [10]. The findings show that torticollis/torticaput is the most prevalent cervical dystonia subtype, while the trapezius, levator scapulae, and splenius capitis were the most frequently injected muscles. Head tremor affected roughly half of the patients where data were available. The disease duration varied, with A Kreisler, 2020 [9] showing a long duration (18.6 ± 11.9 years) compared to the broader range observed in Máté Szabó, 2023 [10]. The clinical improvement and efficacy of BoNT-A treatment were assessed using diverse methodologies across the included studies, reflecting the variability in the literature on cervical dystonia (CD). Below is a summary of the methods used by each study:

- Kreisler (2020) evaluated efficacy indirectly through the accuracy of needle placement in target muscles using ultrasound guidance. While this study did not directly assess symptom improvement, it hypothesized that accurate injections would improve clinical outcomes, such as muscle relaxation and reduction in dystonia symptoms [9].
- Szabó (2023) employed a combination of descriptive statistics and inferential tests, including Fisher’s exact test and Bonferroni correction, to analyze associations between CD subtypes, injected muscles, and treatment efficacy [10].
- Huang (2015) used the Tsui Scale and the Spitzer Quality of Life Index to quantify changes in dystonia severity and quality of life, both of which are validated tools in CD research [8].
- Cordivari (2006) used several scales to evaluate clinical outcomes, including the MBA3, IPA, EDB, and TWSTRS (Toronto Western Spasmodic Torticollis Rating Scale). These tools assess various aspects of dystonia severity, including muscle spasms, pain, and quality of life, and are widely recognized for measuring BoNT-A treatment efficacy [11].

The diversity in methods for assessing efficacy across the studies presents a challenge for directly pooling the data. Each study used a unique combination of clinical scales, imaging techniques, and statistical models to assess outcomes, which limits the ability to perform direct statistical comparisons. However, these varied methods reflect the complexity of measuring treatment efficacy in cervical dystonia and highlight the need for further standardization in outcome measures for future studies. In addition, patient-reported outcomes (PROs) are becoming increasingly important in clinical trials that assess treatment effectiveness. While traditional clinician-rated scales such as the TWSTRS and Tsui score offer useful clinical insights, they may overlook subjective symptoms like fatigue, discomfort, and perceived disability. Incorporating validated PRO instruments tailored to cervical dystonia could provide a more comprehensive understanding of how patients experience functional improvement and changes in quality of life, adding a critical dimension to evaluating treatment success.

## 4. Discussion

The results of this review suggest that ultrasound guidance significantly improves the accuracy and efficacy of BoNT-A injections while reducing the risk of adverse effects. It provides real-time visualization of targeted muscles, and more precise needle placement, minimizing the likelihood of injecting non-dystonic muscles [6]. EMG guidance has also been shown to enhance injection accuracy and effectively reduce cervical dystonia symptom severity, with minimal reported adverse effects [12]. In contrast, Kreisler describes anatomical or palpation-guided injections as remaining suboptimal, as they rely on external landmarks rather than direct visualization, leading to a higher risk of misplacement and unintended injection into non-dystonic muscles [9]. These findings highlight the importance of image-guided techniques in improving the safety and efficacy of BoNT-A injections for cervical dystonia.

However, the successful and consistent implementation of these techniques in clinical practice hinges not only on their theoretical advantages but also on the practitioner’s proficiency. The advanced skill set required to perform ultrasound and EMG-guided injections introduces a significant learning curve, demanding substantial training and hands-on experience to use effectively. To address this, there is a clear need for structured training programs and standardized protocols that promote consistency across clinicians and institutions. As the demand for high-precision injections grows, efforts to define and implement effective training models have gained momentum, including calls for simulation-based training and formal credentialing systems to enhance practitioner competence and confidence. Accordingly, recent efforts have focused on identifying the most effective formats and delivery methods for such training. Effective training programs emphasize hands-on, practical experience over didactic sessions and may incorporate gamification strategies to enhance engagement. A multimodal approach is recommended, whereby even ultrasound-focused courses also expose participants to EMG and electrical stimulation techniques to ensure clinicians are equipped to perform injections across diverse clinical settings. Cadaveric specimens are considered the gold standard for anatomical and injection training, although prosected limbs and live models may be used as cost-effective alternatives to demonstrate real-time anatomy and facilitate EMG practice. Importantly, structured curricula tailored to the trainee’s background, with clear instructor alignment and adaptable content, are considered core components of effective training [14,15]. Evidence strongly supports the superiority of instrumented guidance over manual palpation: one study showed pain relief was significantly lower in the anatomical landmark group (40.0% ± 22.4%) compared to both ultrasound-guided (81.2% ± 34.0%) and EMG-guided (82.2% ± 10.3%) groups (*p* = 0.001) [16]. Additionally, ultrasound-guided injection accuracy was markedly higher than non-guided injections: 95.8% vs. 54.2% for deep (group B) muscles (*p* < 0.001), and 100% vs. 79.2% for more superficial (group A) muscles (*p* < 0.05) [17]. These findings reinforce the critical value of formal training in guided techniques. To promote consistency and reduce variability in clinical outcomes, expert consensus, reached through a modified Delphi method, has called for the development of standardized training protocols, ideally guided by international best-practice frameworks.

The results from this study align with the existing literature. Accordingly, Schramm emphasized that ultrasound-guided injections improve precision, minimize adverse effects, and enhance overall safety [18]. Similarly, Walter found that ultrasound guidance not only enhances injection accuracy but also improves efficacy and reduces adverse effects [5]. Nijmeijer highlighted the benefits of polymyographic EMG guidance, showing that it significantly reduces symptom severity in cervical dystonia [12]. Conversely, Kreisler noted that anatomy-guided injections may have suboptimal accuracy and suggested that ultrasound may improve treatment outcomes [9]. Furthermore, they stated that anatomical guidance carries a higher risk of injecting non-dystonic muscles. Additionally, a systematic review reported that the additional use of ultrasound following EMG-guided injections eliminated recurrent dysphagia in five patients across 27 sessions, supporting the clinical utility of combined guidance strategies [19]. The overall impression is that image-guided techniques, particularly ultrasound and EMG, improve the precision, efficacy, and safety of BoNT-A injections for cervical dystonia. When treating patients with cervical dystonia, clinicians must consider selecting the most effective injection guidance method for BoNT-A treatment. In this sense, ultrasound guidance appears to be one of the most effective techniques [18]. However, while ultrasound provides superior visualization of anatomical structures, EMG guidance remains the gold standard for precisely identifying dystonic muscles, ensuring targeted and effective treatment [12]. By integrating ultrasound for anatomical accuracy and EMG for functional muscle identification, clinicians can minimize the risk of injecting non-dystonic muscles and improve patient outcomes. An innovative technique combining ultrasound and EMG guidance has recently been described for BoNT injections targeting the deep cervical muscles longus capitis (LoCap) and longus colli (LoCol), which are involved in the anterocaput and anterocollis subtypes of cervical dystonia (CD). In a technical feasibility study, patients were positioned with the head stabilized, and ultrasound was used to map key anatomical landmarks such as the anterior tubercles of cervical vertebrae C5 and C6, sternocleidomastoid muscle, carotid artery, and jugular vein. Two ultrasound-guided approaches, in-plane and out-of-plane, were employed to navigate the needle safely, while a monopolar EMG needle provided both muscle stimulation and injection. EMG confirmed the needle’s placement within dystonic muscles by detecting active muscle signals, offering functional validation alongside the anatomical precision of ultrasound. This combined approach allowed precise localization and safe injection of deep cervical muscles, reducing risks such as carotid artery or thyroid gland injury, which are commonly associated with blind or palpation-guided methods. Importantly, this method improved the likelihood of therapeutic success by accurately targeting muscles responsible for the dystonic posture, which are often missed by traditional techniques. The study concluded that combined ultrasound–EMG guidance is a safe, reliable, and effective technique for treating complex CD cases involving deep muscles, and recommended clinicians consider adopting this approach to enhance clinical outcomes and reduce procedural complications [20]. However, the requirement for specialized equipment and training currently limits its widespread use, and further research is needed to assess its cost-effectiveness and broader clinical impact.

One of the key strengths of this review is the rigorous selection process, with strict inclusion and exclusion criteria ensuring that only studies discussing methods of BoNT-A injection were included, excluding any pediatric studies and case reports or case series. Given that cervical dystonia treatment remains an under-researched area, this review provides valuable insights that may help clinicians access relevant data more easily. Additionally, by comparing multiple injection guidance methods within a single paper, this review offers a comprehensive overview of the advantages and disadvantages of each approach, aiding clinical decision-making. However, this review is not without limitations. The strict inclusion of only primary research studies inevitably limited the number of eligible papers and led to a smaller overall sample size. Nonetheless, this narrow focus enhanced the methodological rigor of the synthesis and ensured that the conclusions drawn were grounded in high-quality, directly sourced data, thereby strengthening the internal validity of our findings, even if it may limit their broader generalizability. In addition, heterogeneity among the included studies represents an important methodological limitation. The studies varied significantly in design, patient populations, injection protocols, and, most notably, the outcome measures used. Some assessed objective parameters such as needle placement accuracy, while others used subjective clinical scoring tools like the Tsui Scale, the Spitzer Quality of Life Index, MBA3, IPA, EDB, and TWSTRS. This inconsistency complicates direct comparisons and limits the ability to conduct a meta-analysis. Furthermore, practitioner experience and targeted muscle selection were not uniformly reported, adding further variability. As a result, while overall trends could be observed, drawing definitive conclusions about the superiority of one technique over another remains challenging. This highlights the need for future studies to adopt standardized outcome measures and clearly defined protocols to enhance comparability, reproducibility, and clinical relevance. Establishing uniform assessment criteria for injection accuracy, symptom severity reduction, and adverse effects will improve the reliability of future research and provide clearer guidance for clinical practice. Despite these limitations, this review provides a valuable foundation for future research and clinical practice improvements in the treatment of cervical dystonia. Moving forward, high-quality randomized controlled trials (RCTs) with standardized outcome methodologies are essential to strengthen the evidence base. While ultrasound and EMG have demonstrated significant benefits, further exploration of emerging imaging modalities, such as MRI or high-resolution real-time fluoroscopy, may offer new avenues for optimizing BoNT injections. Investigating these advanced techniques could lead to greater precision, improved efficacy, and reduced adverse effects, ultimately refining the standard of care for patients with cervical dystonia (Table 6).

Cost-effectiveness is increasingly critical when selecting injection guidance techniques for cervical dystonia. Although ultrasound- and EMG-guided injections involve higher initial costs, primarily due to equipment, maintenance, and specialized training, they may prove more economical over time by reducing treatment failures, minimizing the need for repeat injections, and lowering adverse events such as unintended toxin spread. For example, Tyślerowicz et al. (2022) reported that only the ultrasound-guided group showed a significant decrease in TWSTRS disability and pain subscales, indicating superior clinical efficacy compared to other methods [21]. Although comprehensive cost-effectiveness analyses remain scarce, such improvements in clinical outcomes suggest that the upfront investment in guided injection techniques can be offset by long-term benefits. To inform evidence-based reimbursement policies and optimize resource allocation, large-scale health economic studies directly comparing costs, clinical outcomes, and long-term resource utilization across different guidance methods are essential.

Finally, patient preferences and expectations play a crucial role in guiding treatment decisions and should not be overlooked. While some patients may prioritize minimizing side effects or discomfort, others might place greater value on rapid symptom relief or improved daily functioning. Engaging in shared decision-making, discussing the available injection techniques, their advantages, limitations, and supporting evidence, can lead to higher patient satisfaction and stronger adherence to long-term management plans. A truly personalized approach should consider not only the clinical subtype but also the individual’s treatment goals, supporting a more comprehensive and effective care strategy.

## 5. Conclusions

The injection technique of BoNT-A for cervical dystonia remains highly dependent on physician preferences. Nevertheless, the findings from this review suggest that ultrasound-guided injections may provide superior accuracy and better targeting of dystonic muscles. In contrast, EMG guidance appears to offer greater diagnostic value by helping distinguish dystonic from non-dystonic muscles, rather than improving injection precision itself. The anatomy-guided approach remains the simplest and most accessible technique but lacks the accuracy afforded by ultrasound. To establish clearer clinical guidelines and optimize treatment outcomes, further well-designed, high-quality studies comparing these techniques are essential. Such research should aim to standardize outcome measures and evaluate both efficacy and practical considerations to inform evidence-based practice.

## Figures and Tables

**Table 1:** Inclusion and exclusion criteria.

Inclusion Criteria	Exclusion Criteria
Studies involving adult patients diagnosed with cervical dystonia.	Pediatric populations.
Use of BoNT-A (onabotulinumtoxinA, AbobotulinumtoxinA, IncobotulinumtoxinA).	Case reports and case series.
Original research studies (prospective, retrospective, meta-analyses).	Studies not reporting outcomes related to injection techniques.
Studies evaluating or comparing specific injection methods.	

**Table 2:** Description of the various types of dystonia and patient distribution.

Subtype	Description	Features	% of Patients
Torticollis/Torticaput	Neck or head rotates or twists to one side	Asymmetry, SCM, splenius capitis, trapezius; spasms, pain	49.79%
Laterocollis/Laterocaput	Neck or head tilts to one side	Shoulder asymmetry; SCM, splenius capitis, trapezius	17.99%
Retrocollis/Retrocaput	Neck or head forced backward	Balance issues; splenius capitis, trapezius, posterior cervical muscles	8.79%
Antecollis	Neck bends forward	SCM and anterior scalene overactivity; dysphagia	2.09%
Other/Mixed	-		21.34%

**Table 3:** Summary of the data in the included studies.

Author and Year	Study Design	Sample Size	Injection Method(s)	Comparator	Efficacy Outcomes	Safety Outcomes	Injection Accuracy
J H Lee, 2010 [13]	Cadaveric study	20	Anatomical landmarks	-	Optimal injection sites: MEPs 20–40%, IMPs 20–70% from mastoid process	Caution required when injecting SCM due to proximity to nerves/vessels	Ultrasound guidance recommended in 20–40% region
Carla Cordivari, 2006 [11]	Prospective cohort study	20	EMG- guided	BTXA antibody status	45% ≥30% improvement; 80% improvement in antibody-negative; 90% poor response in antibody-positive	Mild, temporary discomfort; no severe reactions	EMG reduces misplaced toxin risk
S W R Nijmeijer, 2012 [12]	Systematic review	40	Polymyographic EMG	Conventional BoNT injection	TWSTRS improvement 27–44%; 70–80% subjective response	Dysphagia (10–14%), mild and reversible weakness	EMG improves targeting accuracy
L Huang, 2015 [8]	Randomized controlled trial	105	Ultrasound- guided	Conventional injection or meds	Group C (ultrasound + braces): Tsui 19.1→3.2; Spitzer 2.8→8.3	Local reactions, temporary weakness	Ultrasound enhances precise targeting and avoids nerves/vessels
Máté Szabó, 2023 [10]	Retrospective cross-sectional study	58	Ultrasound- guided	Manual/EMG-guided	OnaBoNT-A: 117 ± 38.5; IncoBoNT-A: 118 ± 29.8; AboBoNT-A: 405 ± 162 units	Risk of damaging critical structures	High reproducibility with COL-CAP-guided injections
Uwe Walter, 2014 [5]	Literature review	–	Ultrasound- guided	Manual/EMG-guided	Improved outcomes in CD, sialorrhea, spasticity	Risk of ineffective treatment or bruising	High anatomical precision with ultrasound
A Kreisler, 2020 [9]	Randomized controlled trial	56	Anatomy- guided	–	Suboptimal efficacy compared to ultrasound	Minor complications	Ultrasound is better than anatomy-guided

**Table 4:** Data on injected muscles and patient distribution.

Muscle Targeted	% of Patients
Trapezius	56.9%
Levator scapulae	51.7%
Splenius capitis	48.3%
Sternocleidomastoid	32.8%
Semispinalis capitis	22.4%

**Table 5:** Dosing data from Máté Szabó, 2023 [10].

Drug Used	Dose Used
OnabotulinumtoxinA	117 ± 38.5 units (range: 50–180)
IncobotulinumtoxinA	118 ± 29.8 units (range: 80–180)
AbobotulinumtoxinA	405 ± 162 units (range: 100–750)

**Table 6:** Summary of advantages and disadvantages of different injection methods.

Method of Injection	Advantages	Disadvantages
Ultrasound-guided	Enhanced accuracy, avoids vessels/nerves, real-time muscle view, high reproducibility	Expensive; special training; risk of wrong muscle ID or toxin misplacement
EMG-guided	Reduces injection into non-dystonic muscles; improved targeting; lower cost than ultrasound	Requires training; mild discomfort; no anatomical visuals
Anatomy/palpation-guided	Inexpensive; minimal training needed	Only for superficial muscles; no depth info; less accurate
